# The Determination of Predictive Construct of Physical Behavior Change on Osteoporosis Prevention Women Aged 30-50: A Trans-theoretical Method Study

**DOI:** 10.5539/gjhs.v8n3p183

**Published:** 2015-07-27

**Authors:** Farideh Malekshahi, Alireza Hidarnia, Shamseddin Niknami, Frakhondeh Aminshokravi

**Affiliations:** 1 Department of Health Education, Faculty of Medical Sciences, Tarbiat Modares University, Tehran, Iran

**Keywords:** osteoporosis, trans-theoretical model, self-efficacy, prevention, physical activity, women

## Abstract

Osteoporosis is a major public health priority in Iran and throughout the world. The prevention of osteoporosis has recently become the ultimate goal of many health professionals. Behavior change is one of the most powerful strategies to prevent osteoporosis. This study aimed to determine the predictive construct of physical preventive behavior of osteoporosis in women aged 30-50 in Khorramabad, west of Iran. This study included 269 women selected from all the health centers of Khorramabad city according to the inclusion criteria of the study and through random cluster and systematic sampling. The data gathering tools were valid and reliable questionnaires of demographic information, stages of change, decisional balance, self-efficacy, and physical activity. Data were analyzed using descriptive and inferential statistics. The mean of the subjects’ age was 38.72±7.003, and the mean of light weekly physical activity was 38.83±56.400. The results showed that the construct of self-efficacy had the highest predictive power of the preventive behavior. The results also showed that self-efficacy among the constructs of the Trans-theoretical Model was the only predictive construct for osteoporosis prevention behavior. Therefore, the findings of this study can serve as a base for educational interventions in behavioral changes to prevent of osteoporosis by health authorities.

## 1. Introduction

Osteoporosis is the most common type of metabolic bone disease in which bone mass reduces with age. This disease has been known as an important health priority in community public health, particularly in women (Hazavehei et al., 2007; Tussing & Chapman-Novakofski, 2005). The disease remains asymptomatic until bone fracture, and therefore it is also referred to as “silent thief” (Chan et al., 2007). The prevalence of osteoporosis varies among countries and even within countries (Hazavehei et al., 2007). Previous studies have shown that the prevalence of osteoporosis in Iran is mostly at the age of 50 or over, 22.2% in females, and 11% in males (Adib & Nauroy, 2011; Salehi et al., 2009). A fracture due to osteoporosis happens every 3 seconds, and a fracture in the spine occurs every 22 seconds worldwide (PDO, 2006). So that one out of two women over the age of 50 experiences vertebral fractures, and one out of three men over 50 experiences hip fractures in their lifetime, and both of these conditions lead to significant morbidity and mortality (PDO, 2006; Schwellnus et al., 2011). According to global statistics, Iran, like other countries, will have a considerable population of the elderly the next 50 years, so that 14% of the population (11 million people) are 50 or over and 3.6% (2.6 million people) 70 or over, and around 34% of the population (42 million people) will be 50 and over by 2050 (Adib & Nauroy, 2011). The costs of the disease will be enormous (Khorsandi et al., 2011; Moayyeri et al., 2006) so that the medical cost of osteoporosis-related fractures has been more than 22 billion dollars in the USA (Blume & Curtis, 2011). If effective preventive measures are not taken and consistently promoted, it is predicted that the cost of osteoporosis will rise to 200 billion dollars in the world before the year 2040 (Blume & Curtis, 2011). Osteoporosis prevention and its subsequent fractures is the objective of many healthcare professionals (Tussing & Chapman-Novakofski, 2005). According to researches, obtaining a high bone density and maintaining it during lifetime play a major role in preventing osteoporosis in old age (Heaney & Weaver, 2003). Studies have shown that physical activity plays an important role in the prevention of osteoporosis (Lowe et al., 2011; Sharma et al., 2010; Khawaji et al, 2010; Khorsandi et al., 2011). Regular physical activity can have a positive effect on bone mass during lifetime (Alev & Yurtkuran, 2003). Physical activity has obtained a special place in the context of medical science as a therapeutic method (DWS, 2005). Therefore, physical activity, particularly if it is accompanied by weight bearing, results in maintaining and even increasing bone mass through exerting mechanical pressure on bones (Maimoun et al., 2005; Habibzadeh et al., 2009; Ebrahim et al., 2010). Moreover, since behavior change is the basis and foundation of preventing many health-associated risks (Rollnick et al., 2007), so it has been recommended as an effective way to prevent osteoporosis (Karimzadeh Shirazi et al., 2007; Glanz et al., 2008). Experts believe that the efficacy of health education and behavior change programs depends largely on the use of models and theories of health education (Butler, 2001; Araban et al., 2013). The first step of each health education is to choose suitable model for health education (Glans et al., 2008). On the other hand, since the prevention of behavioral factors influencing osteoporosis requires individuals’ behavioral change (Glanz et al., 2008; Araban et al., 2013), the models and theories of health education and health promotion can be effective in designing interventions in this field at different levels (level 1, level 2 or even level 3) of prevention (Parker et al., 2004; Araban et al., 2013). A health education model which can be used to change behavior at individual level is the Prochaska Trans-theoretical model (Prochaska et al., 1992; Prochaska & Marcuse, 1994). The model has been used for a vast range of health behaviors since its introduction (Araban et al., 2013; Salehi et al., 2010; De Vet et al., 2007; Schüz et al., 2009; De Vet et al., 2008; Marcus et al, 1994; Prochaska & DiClement, 1983; Lawrence et al, 2005), this method can predict the way and time of behavioral change (Sharma & Romans, 2008).

**Figure 1:**
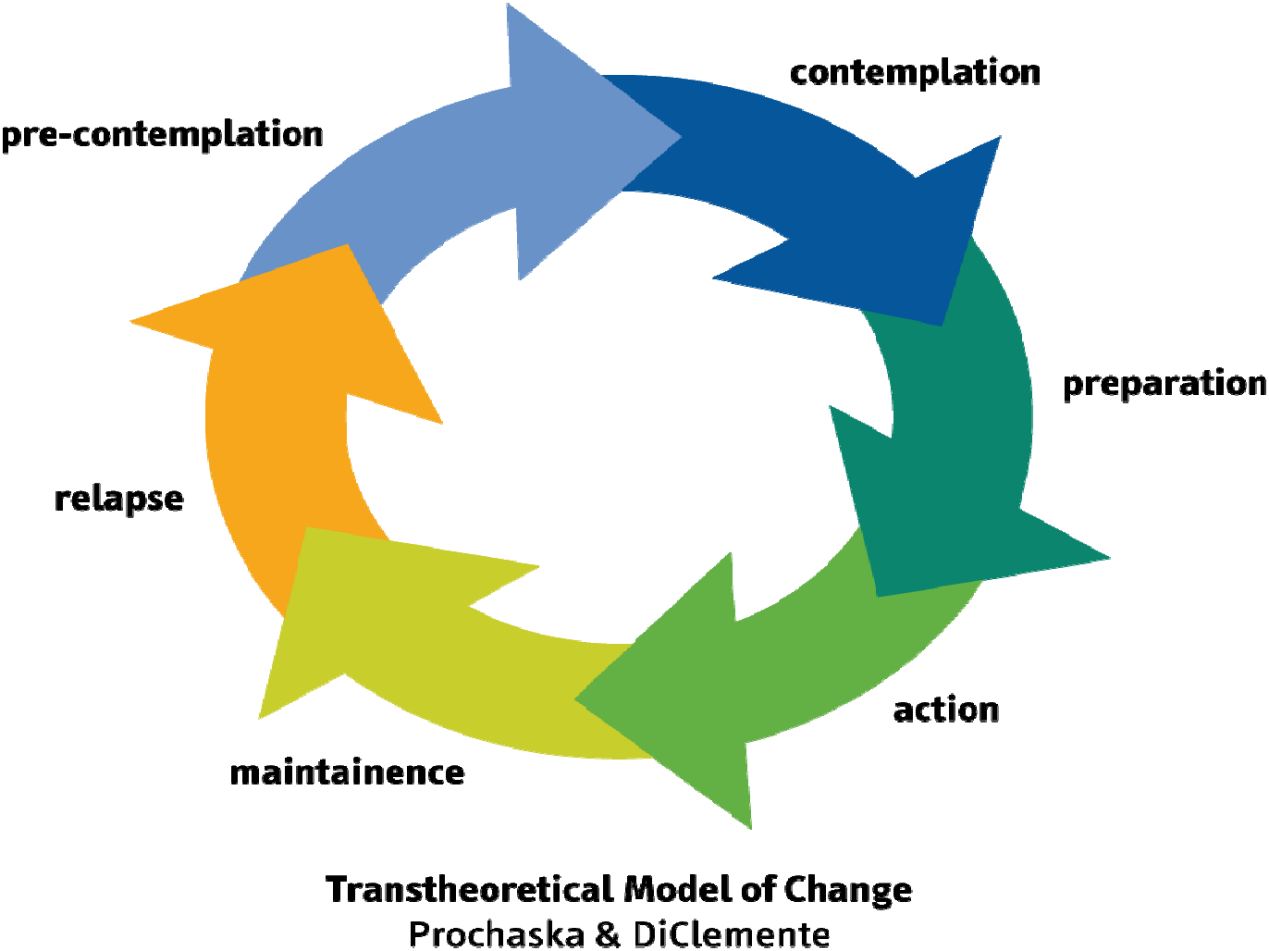
Stages of change in the TTM

The model has four constructs including “stages of change”, “process of change”, “self-efficacy”, and “decisional balance” (Velicer et al., 2001) The construct of “stages of change” has been recognized as a comprehensive model of behavior change. This model focuses on an individual's preparation or effort to change and progress towards healthy behaviors. The importance of the model is for the reason that it shows the occurrence of change in the five stages (Parker et al., 2004) and suggests that people need different interventions at any stage (Figure 1). The first stage is “pre-contemplation” in which the individual does not speculate about behavior change within 6 months. Two groups of people fall into this stage: first, people who are unaware or have little information, these people are not aware of the consequences of behavior; second, those who have experienced change but have failed in the past, these people are resistant to change. The second stage is “contemplation” in which the individual thinks about behavior change in the future (within 6 months). People in this stage will weigh the benefits (pros) and the costs (cons) of behavior change and hence stay longer in this stage. The third stage is “preparation” in which the individual thinks about plans for behavior change in the near future and usually the next month. People in this stage have some plans for change and have taken actions. The next stage is “action” in which the individual has changed behavior during the last 6 months, and new behaviors can clearly be observed in this stage. The last stage is “maintenance” in which the individual has taken actions to maintain behavior change for a long time (6 months or more) (Glans et al., 2008). People in each stage require different interventions, and this categorization will enable us to intervene with regard to the stage which shows how a behavior changes in each of the stages. The processes of change include activities and strategies that will help the individual to progress in the change stages, and include two main types of cognitive processes (related to the individual's thoughts and feelings about unhealthy behavior) and behavioral processes (causing changes in unhealthy behavior). The application of these processes results in strengthening the individual in adopting new behavior and maintaining the behavior (Lee, 2004).

The third construct of the model is “decisional balance”, which is defined as the benefits (pros) and costs (cons) of behavior change from the individual's view (Prochaska & Velicer, 1997; Yalcinkaya-Alkar & Karanci, 2007). According to this model, change occurs when the benefits are greater than the costs. This model is particularly important in the pre-contemplation and contemplation stages (Yasin et al., 2011; Prochaska et al., 2008), and the benefits and costs are similar to those in the health belief model (Prochaska & Velicer, 1997).

The fourth construct of the model is “self-efficacy”, which refers to the individual's perceived confidence in the ability to perform a certain behavior successfully. The construct has been adopted from Bandura's social learning theory and Shiffman's model of consistency and relapse (Bandura, 1982, 1997, 2006). This construct is unique to the relevant behavior and has a pivotal role in changing behavior and moving toward higher stages of change. It also has a direct linear correlation with the stages of change. In this regard, some strategies have been recommended to enhance self-efficacy such as breaking the behavior into small, simple, and practical stages, taking advantage of persuasion, reducing stress, and using believable role patterns (Sharma & Romans, 2008).

This construct has a central role in changing behavior and entering the higher stages of behavioral change (Prochaska et al., 2008). Finally, the model usually interprets behavior change as an event, and evaluates overt behaviors in the end points.

In order to modify osteoporosis prevention behaviors such as regular physical activity that requires behavioral continuity unlike simpler and one-step behaviors, it is logical to apply those models which are sensitive to different stages and small steps of change (Araban et al., 2013). Therefore, the present study aimed to determine the predictors of behavior change in osteoporosis prevention in women aged 30-50 years in the city of Khorramabad (west of Iran). It is hoped that theory-based interventions are designed through identifying the factors influencing the behavior in order to take an important step in improving women's health.

## 2. Materials and Methods

This descriptive-inferential cross-sectional study included 269 women aged 30-50 years old referred to the health care centers of Khorramabad, west of Iran in 2013. The sample size was determined based on the number of the constructs of the Trans-theoretical Model. Therefore, 50 samples selected for each construct, and 7% was added (Hajizadeh & Asghari, 2011). Sampling was performed through the multi-stage cluster sampling. The city was divided into four areas of north, south, east, and west. Then, one health care center was chosen randomly from each area, and the samples were selected randomly from the individuals admitted to the health care centers considering the inclusion criteria of the study. The inclusion criteria of the study were as follows: females aged 30-50 years old with educational level at least grade five of primary school, lack of rheumatoid disease and mental disorder, lack of fractures, pregnancy, menopause and breast-feeding, and consent to participate in the study. Before entering the study, the participants were provided with the necessary information about the study, and those who showed their willingness to participate through verbal informed consent were included in the study. The study was approved by the ethics boards of Tarbiat Modares University and Lorestan University of Medical Sciences. To collect the demographic information, a questionnaire of demographic information was applied, which included age, educational level, marital status, number of children, housework, menstrual age and abnormality, income, height and weight, and tea as well as supplements consumption.

The stage of change in physical activity was measured using the Stages of Exercise Change Questionnaire (SECQ) a 5-item questionnaire, prepared by Marcus et al (Marcus BH et al., 1992). The questionnaire was applied after its content validity index (> 0.7), content validity ratio (0.59), and face validity (IF=5) were approved. The questionnaire put individuals in the stages of pre-contemplation (not thinking about behavioral change in osteoporosis prevention in the next 6 months), contemplation (thinking about behavioral change in osteoporosis prevention in the next 6 months), preparation (thinking about behavioral change in osteoporosis prevention in the next month), action (taking action to change in osteoporosis prevention in the previous day to 6 months), and maintenance (sustaining action in osteoporosis prevention for more than 6 months) (Sharma & Romans, 2008). Since the questionnaire was dynamic in nature, it had one question at five levels. Therefore, the common methods for determining reliability were not appropriate for the questionnaire (Araban et al., 2013). However, since the process of validity confirmation was performed carefully, we can say that the questionnaire had the necessary reliability and validity. The questionnaire placed the individuals in one of the five stages. Therefore, a score of 1 to 5 was assigned to each stage. The preventive behavior in this study was defined as physical activity at least 5 days per week and for 30 minutes each time.

The questionnaire by Nigg et al. (1998) was applied to determine decisional balance (pros and cons), and Schowarzer and Renner's 5-point Health–specific Self-efficacy Scale to measure individuals’ confidence in overcoming the current cons in performing regular physical activity. It should be noted that in this study one question was added by the researchers to the self-efficacy scale, and the decisional balance (pros and cons) scale included 11 questions for each variable equally (Karimzadeh Shirazi et al., 2007). Each question was scored based on a 5-point Likert scale ranging from 1 (*extremely important*) to 5 (*completely unimportant*). The total scores of the questionnaire for the constructs of pros and cons in decisional balance did not range from 11 to 55. The content validity for the pros (CVI=0.99, CVR=0.8), the content validity for the cons (CVI=0.88, CVR=0.70), and the face validity (IF=1.5) were approved. The reliability of these two questionnaires was approved with a Cronbach's alpha of 0.88. The self-efficacy questionnaire included six questions and was prepared based on the theory of Bandura's self-efficacy. The content validity (CVI=0.94, CVR=0.71), the face validity (IF=1.5), and the reliability of the questionnaire (Cronbach's alpha=0.82) were approved. The answers of this tool were on a Likert scale (*“I’m completely sure”* to *“I’m not sure”*) with a range of 6 to 24 scores.

The tool for assessing physical activity was the International Physical Activity Questionnaire (IPAQ). This tool records information on the amount of time spent by a person to walk (as exercise) and to do moderate or high physical activity in the past seven days. The tool was prepared by a group of international experts in 1998 in Geneva, and has already been so far applied in numerous studies, and its reliability and validity have been reported (Craig et al., 2003). The data were collected from the samples in one step and were analyzed with SPSS 16 using descriptive and inferential statistics (Chi-square test, Spearman and Mann-Whitney correlation coefficients). Logistic regression was performed to evaluate the predictive power of the model constructs in adopting the behaviors of osteoporosis prevention, so that the individuals in the stages of “pre-contemplation”, “contemplation” and “preparation” were put into the “non-action” group (those without healthy behaviors), and those in the “action” and “maintenance” stages were included in the “action” group (those with healthy behaviors). The data were analyzed considering the significance level of 0.05.

## 3. Results

The results showed the highest frequency (41%) for the age group of 30-35, the lowest (17.8%) for the age group of 45-50, and the mean age of 38.72±7.003. Moreover, 36.8% had high school diplomas, and 26% had academic degrees. With respect to marital status, 75.1% of the subjects were married. Regarding occupation, 75.1% were housewives, the rest were employed, and 73.1% of the subjects were working in state organizations. Concerning income, low income (less than 500,000 Tomans monthly = 170 USD) with 46.5% and good income (more than 900,000 Tomans monthly = 300 USD) with 20.4% had the highest and lowest frequencies respectively. The mean age of menarche was 13.84±1.9, with the lowest menarche age of 9 (0.4%) and the highest of 21. In addition, the highest frequencies were found for the age of 14 (33.1%) and 13 (21.2%). Also, 11% of the subjects had experienced 1-5 miscarriages. Concerning number of children, 29.4% had two children. In terms of physical activity and housework, 7.8% of the subjects did not do housework, 33.8% did housework for 3 other individuals, and 22.7% for 4 other people. The results also showed that 76.6% of the women had no menstrual abnormalities, and only 23.4% had menstrual abnormalities out of whom 59 (21.9%) had the abnormality of irregularity. The mean and standard deviation of weekly light physical activity was 38.83±56.400 minutes, and the mean and standard deviation of moderate weekly physical activity was 4.77±29.119 minutes (Table 1).

**Table 1:** Demographic Information of Women 30-50 age

	Variables	Percentage
***Age group***	30-35	41
***Marital status***	married	75.1
***Occupation***	housewives	75.1
***Income status***	170USD	46.5
	300USD	20.4
***Menarche age(y)***	< 9	0.4
	13	21.2
	14	33.1
***Number of children***	2	29.4

The results of the construct of “stages of change” related to physical activity in the women showed that 43.5% of the women were in the pre-contemplation stage, and 9.4% in the maintenance stage in performing regular physical activity, as shown in Table 1. Also, in terms of the ranking of stages of change in physical activity, 82.9% were in the non-action phase and 17.1% in the action phase (Table 2). Table 3 shows the results of the constructs of stages of change related to physical activity in the women. Logistic regression was applied to determine the predictors of physical activity behavior (Table 4).

**Table 2:** Frequency distribution of the stages of change related to the variable of physical activity in the studied women based on action and non-action

Ranking of the stages of change	Physical Activity	

	Frequency	Percentage
**Non-action**	223	82.9
**Action**	46	17.1

**Table 3:** Frequency distribution of the stages of change related to the variable of physical activity in the studied women

Stages of change	Physical Activity	

	Frequency	Percentage
**Pre-contemplation**	117	43.5
**Contemplation**	50	18.6
**Preparation**	56	20.8
**Action**	20	7.4
**Maintenance**	26	9.4

**Table 4:** Predictive variables of stages of change related to physical activity behavior based on the Trans-theoretical model constructs

Variable	Confidence interval		Odds ratio	P-value

	Upper	Lower		
**Income**	1.000	1.000	1	0.347
**Educational level**	2.046	0.971	1.409	0.071
**Self-efficacy of physical activity**	1.264	1.083	1.170	0.000
**Pros of physical activity**	1.025	0.933	1.046	0.123
**Cons of physical Coactivity**	1.109	0.988	0.978	0.358
**Occupation**	3.482	0.55	1.384	0.490

Results of the Mann-Whitney test showed a significant relationship between the constructs of stages of change and income (p < 0.006). Also, the findings of Pearson test showed a statistically significant relationship between the constructs of stages of change with educational level (p < 0.043), and occupation (p < 0.014).

## 4. Discussion

The findings showed that only the construct of “self-efficacy” among the applied constructs of the Trans-theoretical Model had the predictive power for the behavior. The results of a comprehensive study by Schwarzer et al. indicated that self-efficacy could have a higher predictive power in health behaviors than the other constructs (Schwarzer & Fuchs, 1996). The results of Berry et al. and Kim's studies on exercise behavior, Swaim's study on the relationship between physical activity self-efficacy and physical activity behavior, and Dishman's et al study reported self-efficacy as the most important predictor of the behavior (Kim, 2004; Berry et al., 2005; Swaim et al., 2008; Gammage et al., 2009; Dishman et al, 2010; Khave et al, 2014). It seems that adopting preventive behaviors for osteoporosis is also more dependent on self-efficacy. Bandura, as the developer of the theory of self-efficacy, believes that self-efficacy is specific to a specific behavior. An individual may have high self-efficacy in one behavior and low self-efficacy in another one (Sharma & Romans, 2008; Bandura, 1977; Kim, 2004; Berry et al., 2005; Swaim et al., 2008; Gammage et al., 2009). Bandura a mentions four factors of success in performance, succession experiences, verbal encouragement, and physiological and emotional arousal as the sources of self-efficacy (Bandura, 2006). Therefore, self-efficacy is an important component of success, which falls in the field of positive psychology (Snyder & Lopez, 2002). The construct of self-efficacy, or belief in “*I can*”, refers to a person's ability to perform tasks in certain circumstances. Additionally, another type of self-efficacy refers to the person's overall belief about his or her own abilities and capabilities (Mazloumi Mahmoud Abad et al., 2010).

Based on the results of our study, 79.9% of the women were in the no-action stage (pre-contemplation, contemplation, and preparation), and 20.1% were in the action stage (action and maintenance) through adopting regular physical activity. Also, most of the women (41.3%) were in the pre-contemplation stage and the lowest number (6.7%) in the action stage, while in Nigg et al.'s study most of the subjects (49.3%) were in the maintenance, the lowest number (2.1%) in the pre-contemplation stage, and 3.8% in the action stage (Nigg & Courneya, 1993). In Mazloumi et al.'s study (2010), 20% of the subjects were in the pre-contemplation stage, 40% in the contemplation stage, 13.6% in the preparation stage, 7.3% in the action stage, and 19% in the maintenance stage (Mazloumi Mahmoud Abad et al., 2010). Therefore, regular physical activity according to regular and specific patterns should be part of an overall strategy to prevent osteoporosis in women, so that an individual can change behavior intentionally (DiClemente, 2005). The results of our study did not show significant differences in the scores of the constructs in terms of marital status. However, Tol et al.'s study indicated that self-efficacy was associated with marital status (Tol et al., 2012). This contrast might be justified according to cultural and social contexts of the studies. Married people spend most of their time on daily life activities, and therefore spend less time on regular physical activity because of the higher responsibilities they have in compares with the other members of their families. However, this interpretation is not always correct. Investigating the relationship between the constructs of the Trans-theoretical Model and regular physical activity revealed that the studied women used more cognitive and behavioral processes in order to adopt physical activity behavior and possessed higher self-efficacy in physical activity when they were in higher stages of change. Based on the Trans-theoretical Model, individuals can go through the stages of change by gaining experience and skills. Returning to the previous stages may even happen, which is justifiable considering the circular nature of the model. The results of our study on decisional balance showed that the central components of pros and cons in this construct are crucial in making decisions on physical activity. The results showed that the individuals in the pre-contemplation stage had negative comments on physical activity such as being time-consuming and tedious (cons), while the women in the maintenance stage had positive comments on physical activity (pros) (Omar-Fauzee et al., 2009; Moeini et al., 2011).

The results showed no significant relationship between the construct of decisional balance and physical activity. This result is consistent with the results of Moeini et al. and Araban et al.'s studies, but inconsistent with the studies conducted by Kidd et al. and Omar-Fauzee et al (Omar-Fauzee et al., 2009; Kidd & Peters, 2010; Moeini et al., 2011; Araban et al., 2013). Therefore, these results suggest that the construct did not have the power to predict the behavior of physical activity. This predictive inability of the pros and cons constructs can be attributed to the high points of these two constructs in the studied population in our study. Therefore, as it is emphasized by Pawlak et al. (Pawlak & Colby, 2009), high scores of pros and low scores of cons cannot predict health behaviors. It is also possible that adopting preventive behaviors (physical activity) for osteoporosis is more dependent on self-efficacy.

The results of the present study showed that, among the constructs the Trans-theoretical Model, self-efficacy is the only construct that can predict the adoption of osteoporosis prevention behavior. Therefore, it is recommended to conduct more extensive studies to confirm our finding. The study did not use the construct of processes of change due to the probable numerous items in the questionnaire in two behaviors of physical activity and calcium intake, and answering the questions took a long time, and these were the limitations of the study. This study is among the first studies carried out on behavior change to prevent osteoporosis in women aged 30-50 in Khorramabad based on the patterns of health education and enhancement.
